# Treatise on Dental Caries

**Published:** 1879-12

**Authors:** 


### BIBLIOGRAPHICAL.

*Treatise on Dental Caries: Experimental and Therapeutic Investigations.*— By Dr. E. Magitot, Laureate of the Institute of France, etc., etc. Translated by Thomas H. Chandler, D. M. D., Harvard. Boston, Houghton, Osgood & Co., 1878.

This volume of 275 pages was received from the publishers some months ago and briefly acknowledged. The illness of the writer prevented at that time the careful reading of the book which it was felt its merits deserved, coming as it does from so distinguished a source; and not until the present has time been found to give it the patient attention it should receive.

This is a small book. Such an one as an American would most probably turn out, as to size, in a few weeks at most. But it bears from beginning to end the marks of that slow, patient, careful procedure which it has seemed to us is characteristic of continental foreign investigation. And this seems to be a merit we are slow to appreciate in our land of hurried ways and premature conclusions. Dr. Magitot leaves no department of the subject of dental caries uninvestigated which is capable of investigation with the lights we have, and at the same time he is modest, yet decided in pronouncing his conclusions.

After a brief statement of his belief as to the origin of dental caries, in which he asserts that in this malady "a chemical alteration" of the associated elements of the tooth takes place, resisted by the vitality of the organ and thus more or less delayed, he summarizes the opinions and beliefs of the earlier writers upon the subject, making mention of the three doctrines held as to the etiology of caries: the inflammatory, the purely chemical lesion, and the compound theory of M. Oudel, of two kinds of caries. He proceeds to divide the work into—

"First, Pathological Anatomy or study of the Lesions of Dental Caries, preceded by a rapid survey of the normal anatomy of the organ.

Second, Etiology and experiments.

Third, Progress, Symtomatology, Complications.

Fourth, Nosology and Diagnosis.

Fifth, Treatment.

Sixth, Finally, after a short resume, we shall give, in the form of aphorisms, a series of conclusions resulting from the whole of our study."

These propositions as introductory, are followed in the first chapter by a resumé of the anatomical structure of the dental organ, exceedingly clear and perspicuous, in which the ground is taken that there is a true "organic movement"—a sort of circulation in the dentine, by which the tooth is nourished. The walls of the canaliculæ have, he states, no trace of organization. The tactile sensibility of the teeth he thinks due to vibration, and not to any peculiarity of structural organization. More stress is laid upon the existence and functions of the membrane of Nasmyth, the "enamel cuticle" of Kolliker, than is common with us. The statement (p. 25) that the dental pulp in old age disappears, requires further proof. It stands here simply as a statement, though the fact may be an exceptional and isolated one. Hypertrophy of the alveolo-dental periosteum in the aged, is given as a cause of loss of the teeth in advanced years.

The discussion of the progress of dental caries is exceedingly careful and discreet, the author showing step by step the various stages with a delightful clearness. The "zone of resistance" is beautifully delineated in the plates which accompany the book. We are, however, not at all satisfied with the cause of the so-called "friction cavities," which Dr. Magitot claims are simply carious spots passed into the condition of spontaneous cure. This, in the location of some of these spots now under observation, is simply impossible, nor is it likely as he claims, that the "entire pulp may disappear under the progressive invasion of the secondary dentine." Stress is laid upon the fact that in active carious conditions, the cavity and its walls have always an acid reaction, while those that have passed into the condition of "dry caries" are alkaline or neutral. No importance is attached to the presence of leptothrix-buccalis in the mouth as a factor in caries; and the grave changes in the condition of the pulp incident to exposure, seem to point to too serious lesions to admit of recovery by treatment or capping.

The second chapter of the book is devoted to, first; the predisposing anatomical conditions and the constitutional causes of caries; second, the role of the saliva considered as the agent of the production of caries; third, the production of artificial caries by direct experiment; fourth, the mechanism of the production of dental caries. In this discussion the anatomical defects of enamel are conspicuously displayed as factors in the production of caries; and indeed, considering the fact that defects of the enamel are apparently wanting in many cases as a cause, this theory is put almost too prominently forward. The elucidation of the causes of erosion is well made, and no one can find fault with the methods by which our author arrives at his scientific conclusions, nor with the law laid down by him, that those teeth are "most predisposed to caries which accomplish their evolution and eruption at that period of infancy most disturbed by diseases, so that in a general way, the conditions of the health always governs, in a certain degree, the constitution of the teeth." The tabulated results of his observations in ten thousand cases of dental caries, are a worthy addition to the statistics of this subject. He finds, however, what does not seem to coincide with common observation in our country, that slight, if any difference exists between the sexes in the proportion of loss of teeth from decay. The influence of drinks, both of water and of acidulated fluids, cider, etc., are discussed with negative conclusions, and heredity is brought forward as an important factor in determining loss.

The best grouping of races we have seen in any book is made by Dr. Magitot, in comparing tendencies to loss of teeth, yet he does not claim that sufficient data are accumulated to base conclusions upon. The conscript tables of France furnish him with figures of value as localizing dental caries, and he seems to find certain districts much more subject to attack than others; but he concludes that the cause is not climatic or due to habit, but to heredity. The Celtic race he claims have good, the Cymric race bad teeth; and much stress is laid on the temperaments thus inherited. No importance is given to the theory that the teeth of pregnant women are robbed of their lime salts to nourish the fœtus. But the doctrine most strongly brought out—*the* doctrine of the book, is the effect of the saliva upon the dental organs. Dr. Magitot is here emphatic even to dogmatism, in asserting that "the saliva is the veritable agent of caries;" and that this agency is due to "the variations its composition undergoes under diverse influences." The section on the study of the saliva is careful, and the veiws seemingly matured with thought from a mass of special experimentations. No one can read this chapter without profit.

But we linger over this book with delight, and must close. The student who takes it up in the hope that it will prove a practical treatise on operations will, of course, be disappointed; but to one who wishes to go back of and beyond the domain of operations into the field of faithful study of the causes of the malady he is daily called upon to treat, cannot fail to be fascinated and charmed as well as instructed.

The table of general conclusions at the close of the book all may not agree with, but that it is the result of a more careful study of this subject than has hitherto been given it, must certainly be granted. The index of illustrations, etc., is very full, and the special index of subjects elaborate. The whole book is a nice one, and we commend it to the students of our colleges who wish something more than the ordinary text-books contain. Dr. Chandler has done his work as a translator quite well, but it is unfortunate that he has translated so literally. The terms resection for removal of superficial decay, obturation for filling, etc., do not add to the value of the book, and sound oddly to American ears. H.

---

*American Academy of Dental Science.*

